# pH-dependent structural diversity of profilin allergens determines thermal stability

**DOI:** 10.3389/falgy.2022.1007000

**Published:** 2022-10-17

**Authors:** Florian Hofer, Anna-Lena Fischer, Anna S. Kamenik, Franz Waibl, Monica L. Fernández-Quintero, Klaus R. Liedl

**Affiliations:** Department of General, Inorganic and Theoretical Chemistry, University of Innsbruck, Innsbruck, Austria

**Keywords:** allergens, profilins, protonation dependence, flexibility, thermal stability

## Abstract

The family of profilin allergens is a common class of proteins found in plants, viruses and various eukaryotes including mammals. Profilins are characterized by an evolutionary conserved structural fold, which is responsible for their cross-reactive nature of Immunoglobulin E (IgE) antibodies. Despite their high overall structural similarity, they exhibit substantial differences in their biophysical properties, such as thermal and pH stability. To understand the origin of these functional differences of Amb a 8, Art v 4 and Bet v 2, we performed constant pH molecular dynamics simulation in combination with Gaussian accelerated MD simulations. Depending on the respective protonation at different pH levels, we find distinct differences in conformational flexibility, which are consistent with experimentally determined melting temperatures. These variations in flexibility are accompanied by ensemble shifts in the conformational landscape and quantified and localized by residue-wise B-factors and dihedral entropies. These findings strengthen the link between flexibility of profilin allergens and their thermal stability. Thus, our results clearly show the importance of considering protonation dependent conformational ensembles in solution to elucidate biophysical differences between these structurally similar allergens.

## Introduction

**Figure 1:**
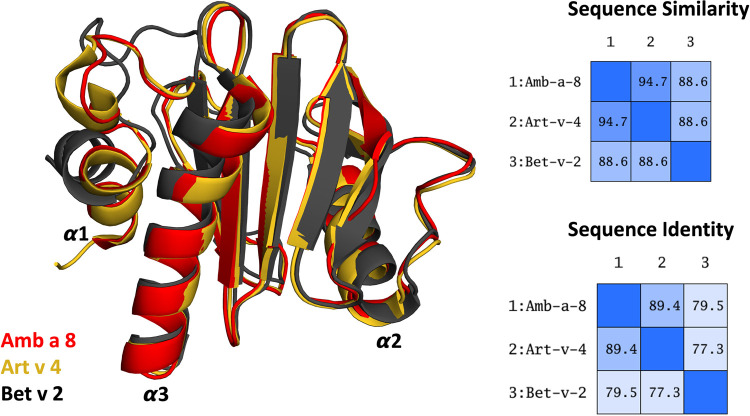
Overlay of the structures of the three simulated profilin allergens. Sequence similarity and identity between the systems is shown on the right.

More than 25% percent of the population are affected by IgE-mediated allergies (1–4). An IgE-mediated allergy is a hypersensitivity disease, which is characterized by the production of IgE antibodies against antigens (i.e., allergens) which intrude into the body. However, despite extensive research efforts, the reasons why some proteins cause an allergic immune response in individuals remains elusive (5, 6). Additionally, it has been shown, that already small variants in sequence and or structure can trigger a completely different immune response (7–13). Thus, understanding or predicting the allergenic potential of a protein based on their sequence and structural similarity is still challenging. Profilins constitute a family of highly conserved proteins, which are also known as panallergens (14, 15). The classification as “panallergen” refers to minor allergens, which exist in most eukaryotic cells, including plants, fungi, protozoa, animals, and viruses, and are responsible for IgE cross-reactions even between unrelated pollen and plant food allergen sources (16). Even though they are considered to be minor allergens, sensitization to panallergens can result in various sensitizations (16, 17). Apart from being ubiquitous, the profilin family is characterized by a highly conserved sequence (approximately 80% sequence identity, Figure 1) and structure, which is a pre-requisite for cross-recognition by IgE. Profilins are responsible for regulating various cellular processes such as membrane trafficking, actin cytoskeletal dynamics or binding to proline-rich regions of proteins (14, 18). Particularly interesting are profilins, which are involved in eliciting seasonal allergies, originating from weed pollens, grass and plants (15, 19). Here, we focus on three profilin allergens, two originating from weeds, namely Amb a 8 and Art v 4 and one from trees, i.e., Bet v 2. Amb a 8 originates from short ragweed (*Ambrosia artemisiifolia*), Art v 4 can be found in mugwort (*Artemisia vulgaris*) (19–22). Both ragweed and mugwort have overlapping flowering periods and are considered the most important sources of profilin allergens. Therefore the differential diagnosis between ragweed and mugwort pollen allergy has been challenging for allergologists in areas where both plants occur (16, 20, 21, 23). The birch pollen allergen Bet v 2 has in fact been the first profilin identified to be a pollen allergen. Bet v 2, despite being a minor allergen, is involved in IgE cross reactivity between plants and food (19, 22). Recently, these profilins have been biophysically characterized and pH dependent melting temperatures have been provided (19).

Several studies have demonstrated that allergen fold stability influences allergic sensitization (24–26). This relation is typically attributed to of T-cell activation following the MHCII pathway (27). Here, the allergens are digested by proteases into small peptides (28). This process is accompanied by a significant drop in the pH, facilitating the destabilization and subsequent unfolding of the allergens. The resulting peptides are then transported to the cell surface and later presented by major histocompatibility complex class 2 molecules to the immune system. The pH stability of an allergen directly influences the kinetics of the proteolytic digestion and in consequence the loading of the MHC class II molecules, which in turn determines the T-cell polarization and thus the immune response itself (25, 26, 29).

This pathway triggers the recognition of allergens after their endosomal uptake and proteolytic degradation. A protein's tendency to unfold under varying pH condition directly relates to the antigen presentation kinetics and thus contributes critically to the resulting immune response (25, 29).

Interestingly, we previously observed already the melting temperatures of allergen proteins can already be a reasonable indicator for their fold stability and proteolytic susceptibility (25, 26).

However, with constant pH MD simulations we can explicitly model allergen dynamics local unfolding during endolysosomal acidification (12).

Thus, in this study, we aim to elucidate structurally and mechanistically the experimentally observed differences in biophysical properties, by characterizing the conformational diversity and fold stability with constant pH simulations and enhanced sampling techniques. This protocol allows us to capture the influence of acidic pH on the dynamics of these allergens.

## Methods

### Structure preparation

Starting structures for the cpH-MD simulations were prepared from the available crystal structures on the protein data bank, using the structures 5EVE (Amb a 8), 5EM0 (Art v 4) and 5NZB (Bet v 2) (30). All residues not corresponding to the actual allergen itself were removed during setup. Topologies and starting coordinates were prepared with the tLEaP module of AmberTools 20 (Case et al.), using the ff99SB force field (31), along with modification necessary for cpH-MD (32–34). Generalized Born (GB) radii of the titratable oxygens in the aspartate and glutamate side chains were reduced to 1.3 Å, as suggested by Swails et al. (34). Each system was soaked in a truncated octahedral box of TIP3P (35) water with a minimum wall distance of 10 Å. All systems were equilibrated with an extensive protocol before production (36, 37).

Starting structures for the GaMD simulations were extracted from the obtained cpH-MD trajectories as follows: For each system, at each simulated pH value, the trajectories were clustered into 5 clusters with the program cpptraj of AmberTools 20 (38) using a hierarchical agglomerative approach and average linkage. Each cluster structure was then set up for subsequent GaMD simulations with the program tLEaP (38) using the ff14SB force field (39) and a cubic TIP3P water box, with 10 Å padding.

### Simulation setup

For all simulations the GPU implementation of the pmemd module of Amber 20 (38) was used. We used a Langevin thermostat with a collision frequency of 5 ps^−1^ to keep the temperature constant at 300 K. During production simulations, a Monte Carlo barostat was used to keep constant pressure of 1 bar using a pressure relaxation time of 2 ps (40). A Berendsen barostat was used during equilibration (41). A non-bonded cutoff of 10 Å was used for the cpH-MD simulations (8 Å for the GaMD simulations) and long-range electrostatics were treated with the Particle-mesh Ewald approach (42). All bonds involving hydrogens were restrained with the SHAKE algorithm (43) to allow for a time step of 2 fs. For the cpH-MD simulations, a salt concentration of 0.1 M was used (34), titrations were attempted every 200 steps, followed by 200 steps of solvent relaxation in case of at least one successful titration. A total of 1 µs of simulation time was collected for each system at each pH value. GaMD simulations (44, 45) were run using the dual boost implementation, the threshold energy was set to its lower bound. The number of steps to update the potential energy statistics was set to four times the number of all atoms in the systems and rounded to the closest multiple of 500, corresponding to ∼150–180 ps with a 2 fs timestep depending on the system. The closest multiple of these steps to 2 and 6 ns was used as equilibration time (using conventional MD simulations) and to update the GaMD acceleration parameters. Finally, 200 ns of production GaMD simulations with the final set of acceleration parameters were collected per cluster, resulting in an aggregate simulation time of 12 µs.

### Analysis

Trajectories were processed and analyzed with cpptraj and pytraj of AmberTools 20, as well as vmd and in-house python scripts (46–49). PCA analyses was done with the PyEMMA package, version 2.5.7 (50). PyMol was used for structure visualization (51). Dihedral entropies were calculated using the X-Entropy package (52). Unless otherwise mentioned analyses were focused on the core of the proteins, excluding the short N-terminal helix and the loop linking it to the protein core. This was done for several reasons. First, in the crystal structure of Bet v 2 this helix shows a noticeable kink, which is not present in the other crystal structures. While the structure of the helix relaxes already at the cpH-MD stage, the kink still renders the helix in the Bet v 2 system more flexible than in the other systems. Second, the linking loop to the protein core is longer in the Bet v 2 system, introducing even more flexibility and making a consistent alignment difficult. Excluding this part in all three systems facilitates a concise definition of the protein core and a consistent alignment.

## Results

### Profilin dynamics at varying pH levels

To assess pH dependent differences in the dynamics of the three profilin systems and obtain a broad conformational sampling, we subjected each system to 1 µs of constant pH simulations at four different pH levels, totaling in 4 µs of cpH simulation time per system. Hereafter, the simulations were geometrically clustered to obtain a highly diverse set of starting structures for the subsequent Gaussian accelerated MD simulations. We considered five clusters per system and pH, which were used as starting structures for GaMD simulations. The obtained cluster representatives were structurally analyzed, with special respect to differences in protonation states obtained from the cpH-MD. Interestingly, we find that despite the observed conformational rearrangements the profilins exhibit consistent protonation pattern within each pH level. However, even at the same pH the three investigated allergens clearly differ from one another in their preferred protonation. Consequently, despite their striking similarity in sequence and structure, Bet v 2, Amb a 8 and Art v 4 strongly differ in the available h-bond networks and overall charge distribution.

**Figure 2:**
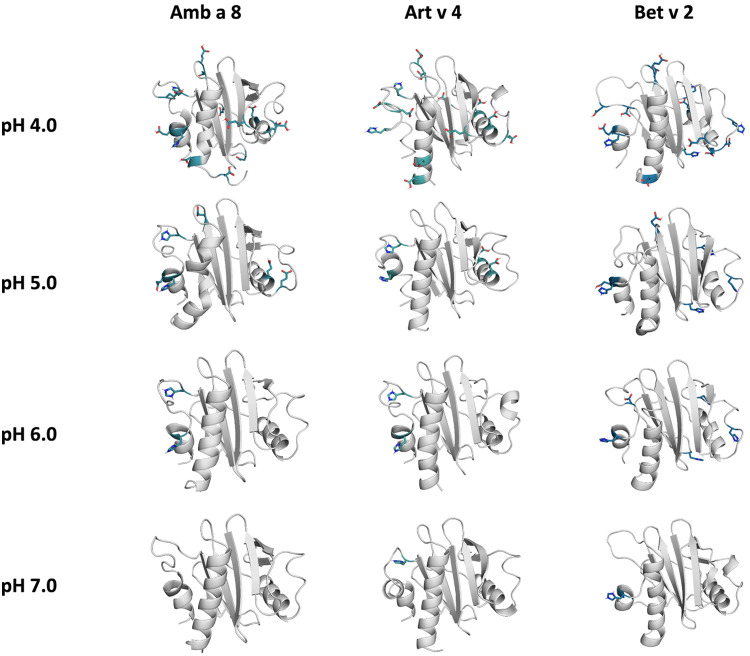
Cluster representatives of the highest populated clusters obtained from the 1 µs cpH-MD simulations. Residues showing differences in protonation are shown as sticks.

The applied technique of constant pH MD simulations allows us not only to investigate in protonation probabilities with high reliability, but also capture differences in pH-dependent conformational dynamics (12, 53, 54). For two of the studied allergen proteins we observe substantial structural rearrangements, i.e., unfolding of the α2 and α3 helix (see Figure 1 for the structure) at low pH in Amb a 8 and Bet v 2 (Supplementary Figure S1). Supplementary Figure S1 shows an overview of all cluster representatives at each pH value for the investigated profilins resulting from 1 µs of cpH MD. A trend towards more diverse structures at lower pH values can be seen for all systems. Furthermore, the structural ensembles of Bet v2 appear to be more diverse across all pH values when compared to the other two systems. To explore the conformational space accessible to each allergen even more exhaustively, these cluster representatives were then used to perform 200 ns of GaMD simulations (Figure 2). The local unfolding of the α2 and α3 helix is also reflected in Figure 2 (at pH 5.0 and pH 4.0).

### Exhaustive exploration of the free energy surface with enhanced sampling

**Figure 3:**
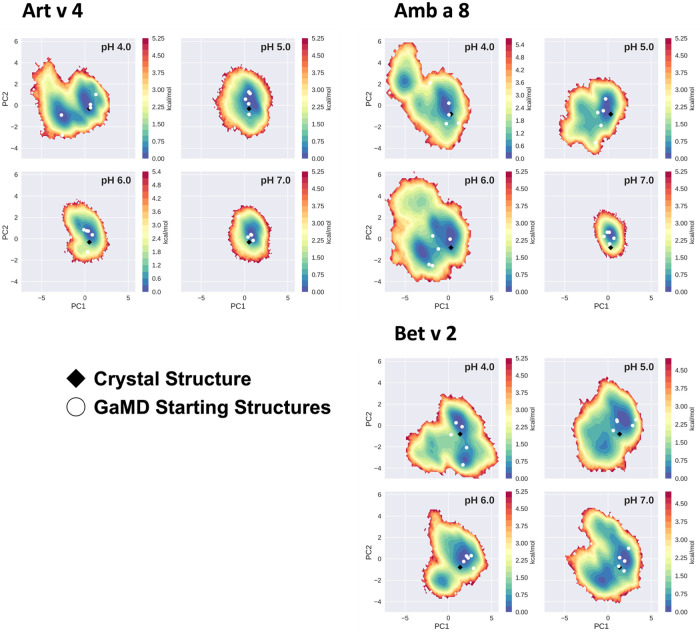
Free energy landscape explored by each allergen. The structural data accumulated for each profilin is projected onto a combined PCA plot to visualize the explored conformational space at each individual pH value. Crystal structures of each system are shown in black diamonds and the GaMD starting structures are depicted as white circles.

The conformational space sampled with GaMD simulations was analyzed and visualized *via* principal component analysis (PCA), see Figure 3. The analysis was based on all trajectories of all systems at all sampled pH values and the individual trajectories were mapped onto the constructed space. For this analysis the coordinates of the core of the proteins were used, as detailed in the "Methods" section. Furthermore, for each system the respective crystal structure was mapped into the space and marked as a black diamond. Comparing the sampled spaces, we find that Art v 4 is notably the most stable of the three simulated systems, showing only minimal fluctuations at pHs 5–7, as is indicated by the single, very deep local minimum in free energy at these pH values (Figure 3). This minimum is structurally also very close to the crystal structure. Only at pH 4 a second local minimum is explored. This is different for Amb a 8 and Bet v 2 (Figure 3). While Amb a 8 still shows only one deep local minimum at pH 7, the sampled space is notably broader at lower pH values multiple local minima are explored. Also for this system, the main minimum is comprised by structures closely related to the crystal structure. On the other hand, the Bet v 2 system already shows a notably higher flexibility at pH 7.0: the sampled conformational space is broader and the minima shallower. The crystal structure of Bet v 2 is located between the two main minima at pH 7, with very low transition barriers in between. Furthermore, the minima encountered of the Bet v 2 system are different than the ones encountered in the other 2 systems. Additionally, Figure 3 shows the seeds used for the GaMD simulations mapped into the combined PCA space. For Art v 4 the seeds are well distributed over the sampled space, however, for the Amb a 8 and especially Bet v 2 we see that a considerable portion of conformational space is explored even where no seed was initially placed. In the case of Bet v 2 this is also the case at pH 7 and indeed no seed was initially located in the second minimum.

### Quantitative differences in fold stability

**Figure 4:**
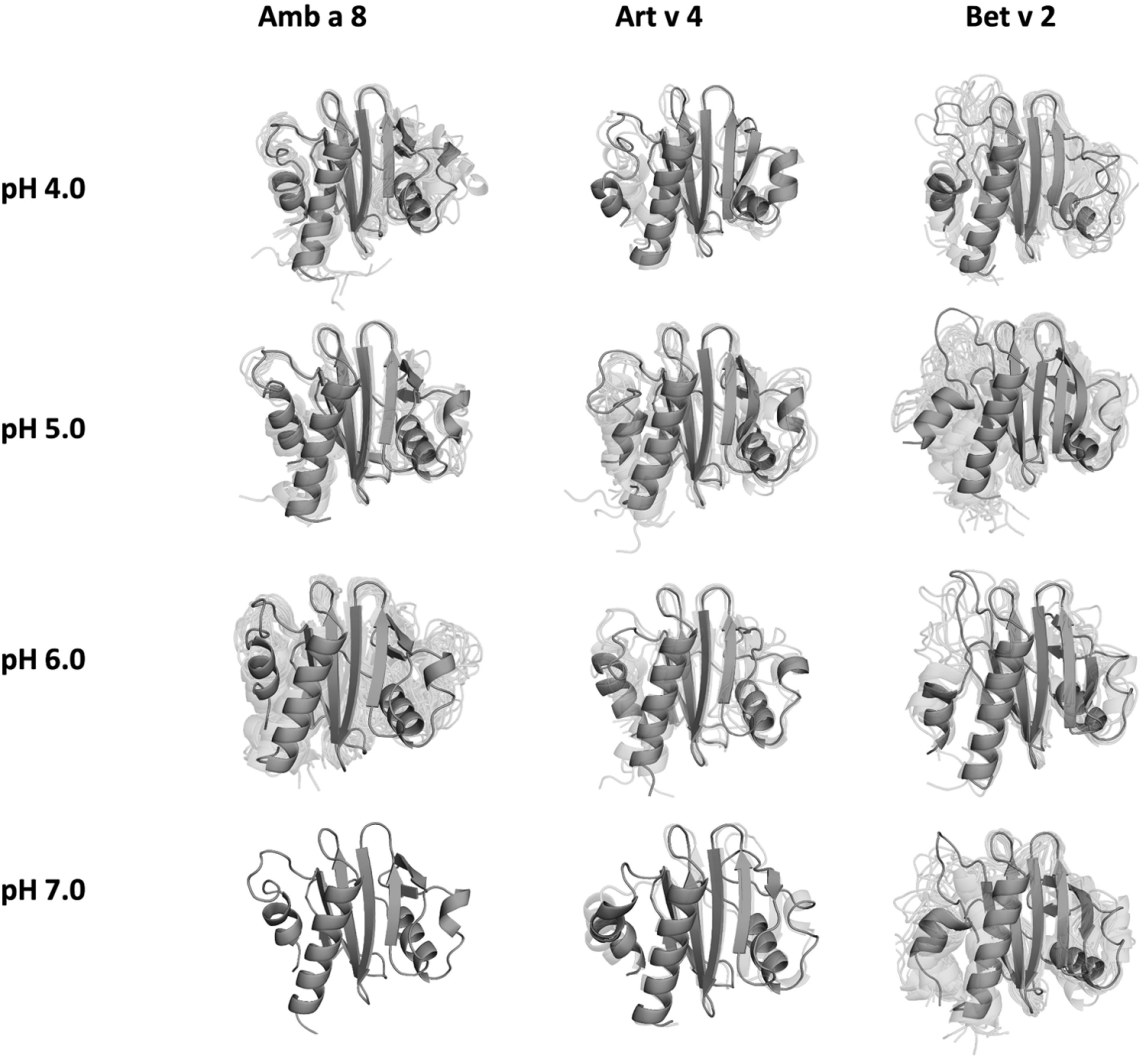
Representative conformational ensembles obtained by clustering the GaMD simulations at different pH values with the same clustering distance cut-off criterion.

**Figure 5:**
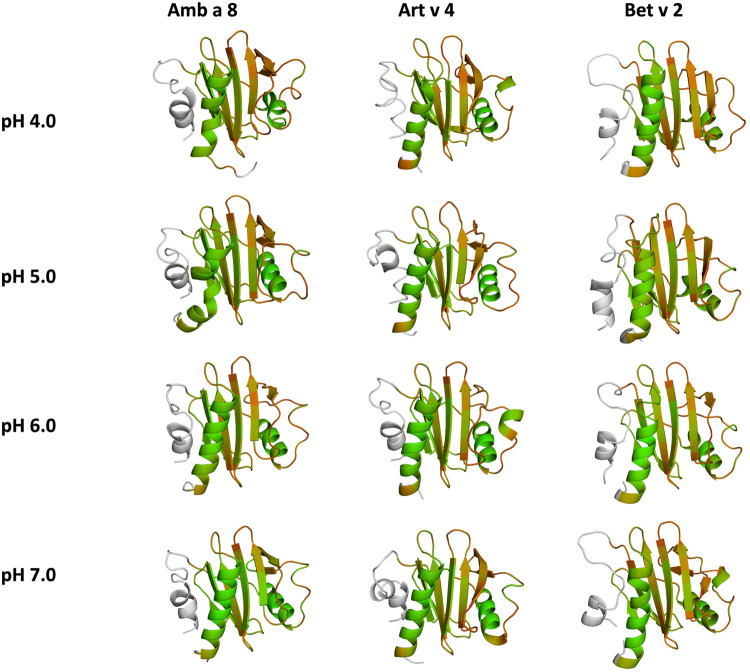
Quantifying the conformational diversity of each system. The overall sum of the residue-wise dihedral entropies at each pH value for all simulated systems is shown as a measure of structural heterogeneity. Higher values of S denote a higher flexibility of the respective allergens. The structures are color-coded based on their entropy value: green (low flexibility, red-high flexibility).

**Figure 6:**
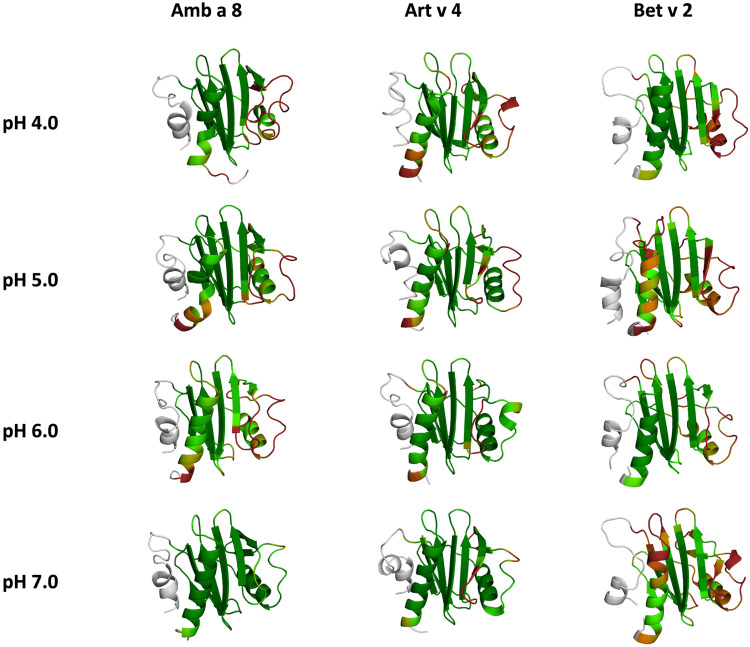
Residues-wise B-factors are mapped onto the structures of the most populated clusters of the 1 µs cpH-MD simulation. The more flexible regions are colored in dark-red, while the more rigid parts of the protein are depicted in dark-green.

In order to quantify differences in the flexibilities of the three systems, we performed a hierarchical cluster analysis and calculated residue-wise dihedral entropies and B-factors. The hierarchical clustering was performed on the backbone atoms of the three profilin allergens, by using the same distance cut-off criterion of 2 Å for all systems at each pH value. We find at each pH level, the highest number of clusters for Bet v 2 (Figure 4). Thus, already when comparing the number of clusters between the variants at different pH values, the differences in dynamics become apparent. To facilitate a per-system comparison, we calculated the overall sum (S) for each system at each simulated pH value, see Figure 5. Hence, a higher value of S signifies a higher diversity of the conformational ensemble, or in other words: lower fold stability. Except for pH 6, the Bet v 2 system consistently shows the highest dihedral entropy of the three simulated systems. Furthermore, we note that for all systems the trend in entropies increases, as the simulation pH decreases. The trend of having a higher flexibility and a lower fold stability is even clearer in Figure 6, where we calculated residue-wise B-factors, projected onto the highest populated cpH MD cluster. Bet v 2 reveals the highest flexibility, followed by Amb a 8. Art v 4 is the most rigid profilin allergen, especially at pH 5. Additionally, in contrast to Amb a 8, Art v 4 retains the native fold. A similar, but slightly weaker trend can be seen in our distance RMSD analysis (Supplementary Figure S2). Especially at pH 7.0 the Bet v 2 system is significantly more diverse than both the Art v 4 and the Amb a 8 systems and retains a similar level of diversity at lower pH values. In contrast, Art v 4 and Amb a 8 show an increase in diversity at lower pH values. This shows does not only show in the median DRMSD values, but also in their broader distributions.

## Discussion

In this study we thermodynamically characterize the conformational diversity of three profilin allergens, namely Art v 4, Amb a 8 and Bet v 2, showing that changes in the protonation at lower pH strongly influence the flexibility and consequently contribute to the loss in thermal stability. This has further relevance in the context of the processing of internalized allergens within antigen presenting cells, as there, the allergens are destabilized by acidification. Differences in pH stability of the allergens influence the kinetics of their proteolytic digestion and in consequence the type of T-cell polarization and the subsequent immune response. These results are compelling, considering the fact, that all three allergens have a high sequence identity and similarity (>80%) and share a near identical fold (Figure 1). Allergen stability is defined as the ability of the proteins to withstand chemical and physical changes in the environment as well as resistance against proteolytic degradation and still retain their native fold. The intact three-dimensional structure of an allergen is a critical determinant for its allergenic potential. Thus, understanding the mechanism of thermal degradation of allergens, and the structural consequences thereof is a crucial aspect to elucidate their role in the immune system (29, 55). The structural and functional changes associated with the melting process of a protein are highly complex and still remain elusive (11, 12, 53, 55). Here, we show that a decrease in thermal stability at varying pH levels is accompanied by an increase in flexibility, which is reflected in higher dihedral entropies (Figure 5), B-factors (Figure 6) and a broader conformational space (Figure 3). In Figure 6 the residue-wise B-factors were mapped onto the structure representatives of the highest populated clusters obtained from the 1 µs cpH MD simulations. The B-factors reveal a clear distinction between the different profilins at different pH values becomes apparent. Most excitingly, already at pH 7 we find that Bet v 2—the allergen with the lowest melting temperature—shows a substantially higher flexibility, especially in the α2 and 3 helices, than the other profilins. This enhanced flexibility of Bet v 2 goes hand in hand with local unfolding events of the α3 helix. Amb a 8 and Art v 4 show a clear difference in the dynamics at pH 5, as the α3 in Amb a 8 starts to locally unfold, while Art v 4 retains its native conformation, which is in line with the experimentally available pH dependent melting temperatures (19, 22). Hence, we suggest that the higher flexibility at lower pH values is a pre-requisite for thermal degradation. The fold stability of the allergens we discuss here will also have an impact on the process of epitope recognition by IgE/IgG, since the epitopes need to be in their native fold. Local unfolding or other major conformational changes as those observed here (Figure 2) would make the IgE recognition impossible if the respective epitopes are destroyed by the structural rearrangements. An example for this would be the α3 helix, which is a known epitope (PDB accession code 7SBG of a structurally highly homologous profilin allergen in complex with IgE) and indeed unfolds at lower pH in the cases of Bet v 2 and Amb a 8 (56).

Additionally, this change in dynamics can also be quantified in the dihedral entropies, however, less pronounced (Figure 5 and Supplementary Figure S3). This trend is not only expressed in the B-factors and the dihedral entropies, but is also reflected in the respective structural ensembles, where we consistently find a higher number of clusters, which implies a broader conformational ensemble, upon a pH induced decrease in stability (Figures 3, 4, Supplementary Figure S2). The importance of considering protonation dependent ensembles in solution, instead of single static structures, becomes even more apparent when visualizing the accessible free energy landscapes (Figure 3). We do not only observe distinct conformational states between Bet v 2 and the other two profilin allergens, but also find a significantly broader and more shallow conformational landscape. This broader conformational landscape in combination with new minima in solution indicate that lowering the pH level already at room temperature destabilizes Bet v 2 significantly more than the other studied allergens. Our findings thus strongly suggest that the differences in protonation patterns at the studied pH levels is the driving force for the observed variation in thermal stability of Art v 4, Amb a 8 and Bet v 2. Furthermore, this study highlights the value and benefits of our applied workflow that involved explicit simulation of protonation probabilities and enhanced sampling. The proposed workflow is transferable and can be applied to many other allergen families, for which thermal and/or pH stability is a key aspect, for instance food allergens.

With the proposed strategy we contribute an atomistic model of allergen dynamics at varying pH levels. These models rationalize the experimentally observed trends in thermal stability, which has previously been established as a central marker to understand immunologic potency of allergen proteins.

## Conclusion

Applying a complementary state-of-the-art MD simulation strategy for three homologous profilin allergens we highlight the subtle, yet impactful differences in their protonation state ensembles at varying pH levels. In particular, by exhaustive sampling of their conformational free energy landscape we were able to quantify how the varying protonation preferences propagate to clearly distinct structural dynamics. Our main finding is that already at room temperature Bet v 2—the thermally least stable profilin—shows the lowest fold stability across all pH values. Hence, we argue that for the studied proteins the differences in protonation play a key role for the observed thermal stabilities. Considering the striking identity of the studied allergens in sequence and structure, our study emphasizes the significance of considering the dynamic nature of allergen proteins in order to understand their biophysical and immunological properties.

## Data Availability

The original contributions presented in the study are included in the article/**Supplementary Material**, further inquiries can be directed to the corresponding author/s.
