# Supplementary material for: pH-dependent structural diversity of profilin allergens determines thermal stability
Source: Front Allergy. 2022 Oct 17;3:1007000. doi: 10.3389/falgy.2022.1007000 (PMC9618696; doi:10.3389/falgy.2022.1007000)
Supplement: Supplementary file 1 [file DataSheet1.docx]

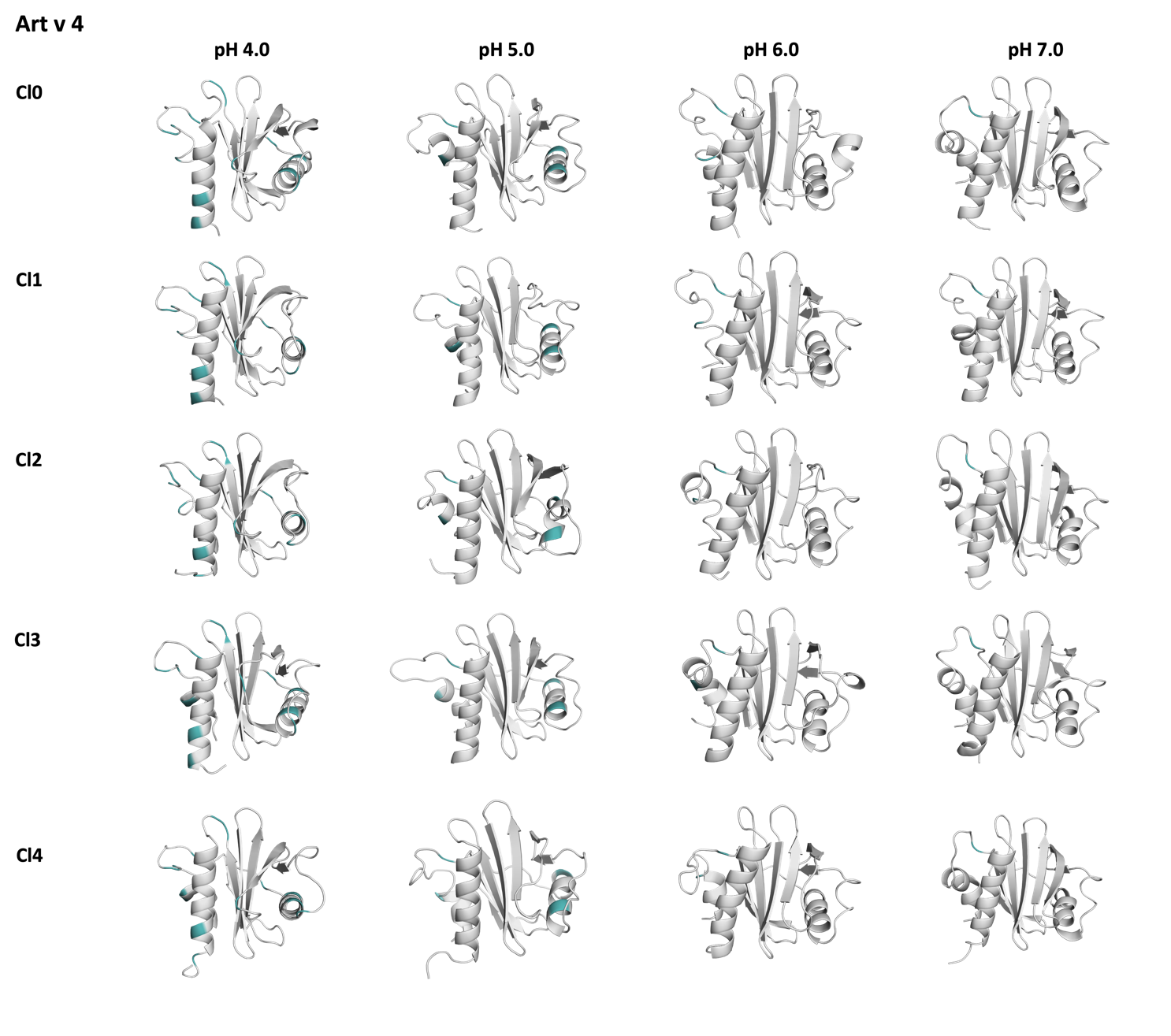


SI Figure S1: Overview of the cluster representatives obtained from the 1 µs of cpH MD for all three profilin allergens, color-coded according to the protonated residues. The clusters are sorted based on their respective probability.


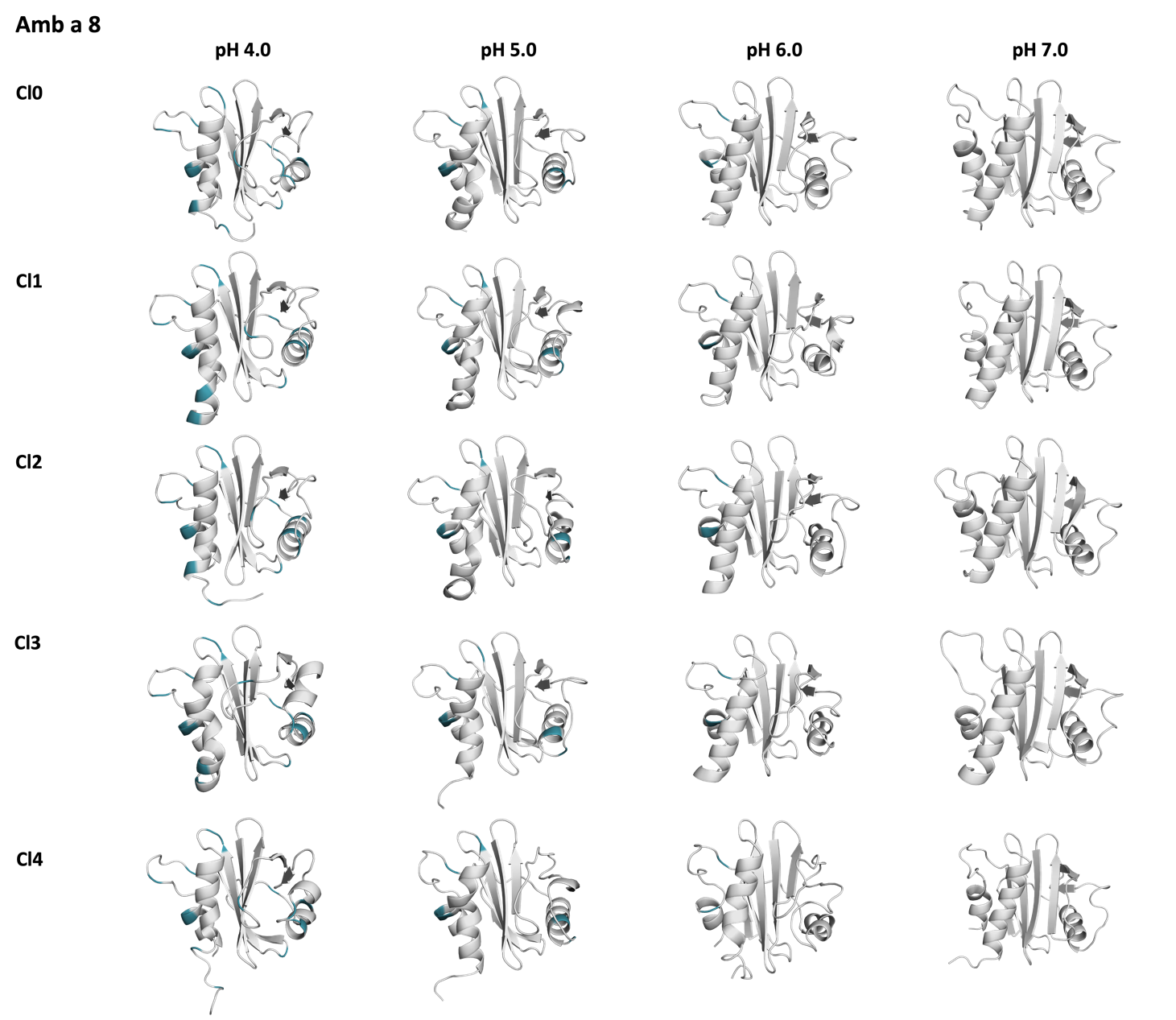
 SI Figure S1: Overview of the cluster representatives obtained from the 1 µs of cpH MD for all three profilin allergens, color-coded according to the protonated residues. The clusters are sorted based on their respective probability.


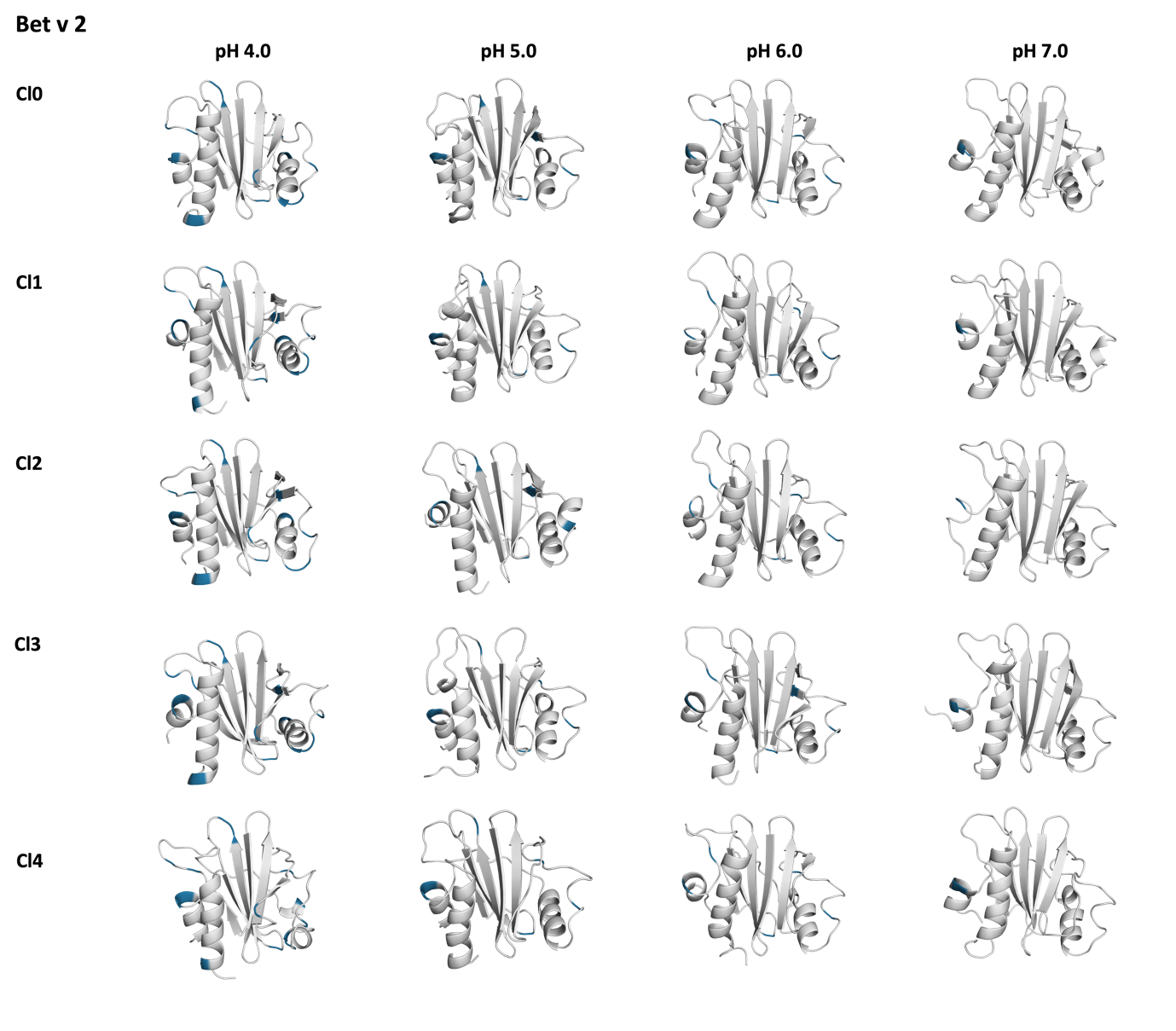


SI Figure S1: Overview of the cluster representatives obtained from the 1 µs of cpH MD for all three profilin allergens, color-coded according to the protonated residues. The clusters are sorted based on their respective probability.


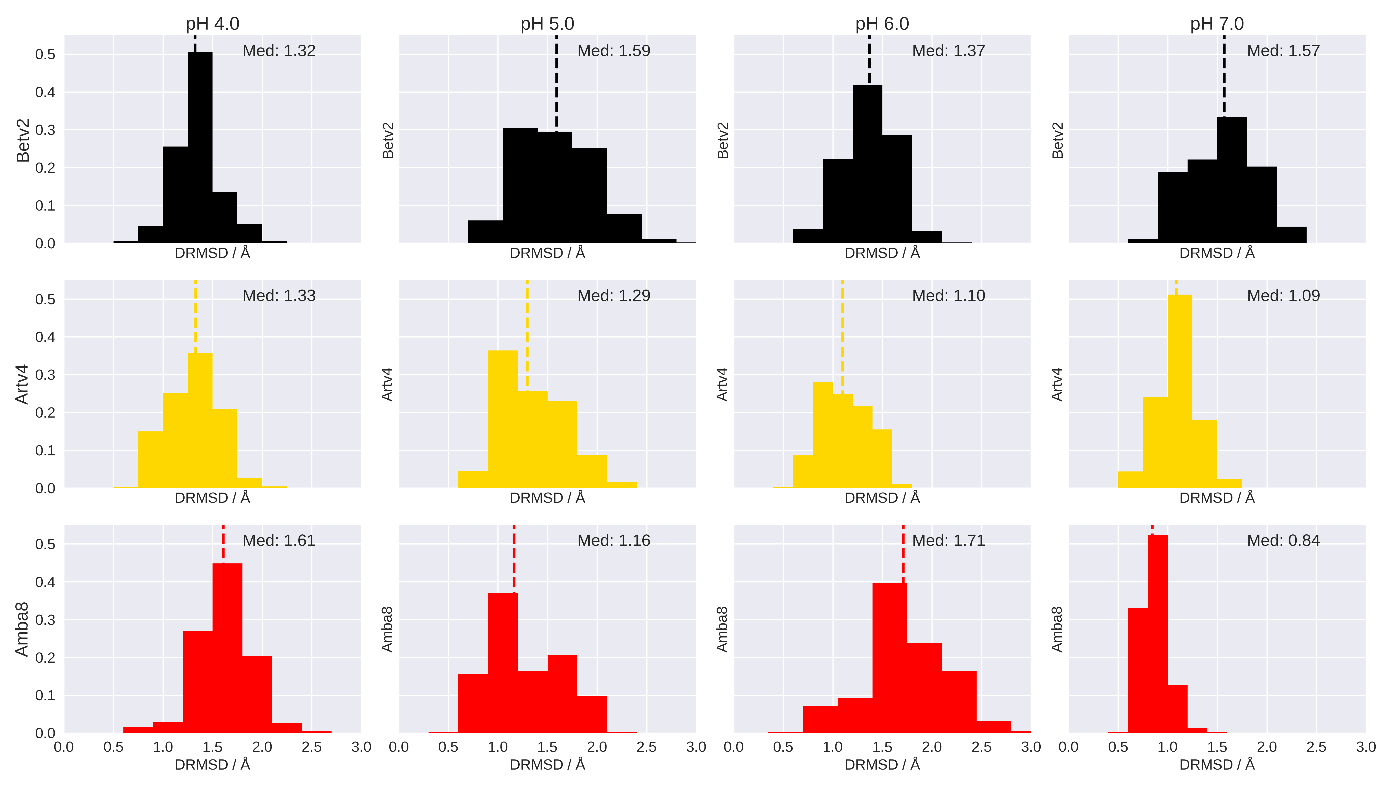


SI Figure S2: DRMSD Plots for the three investigated profilin allergens across all simulated pH values.


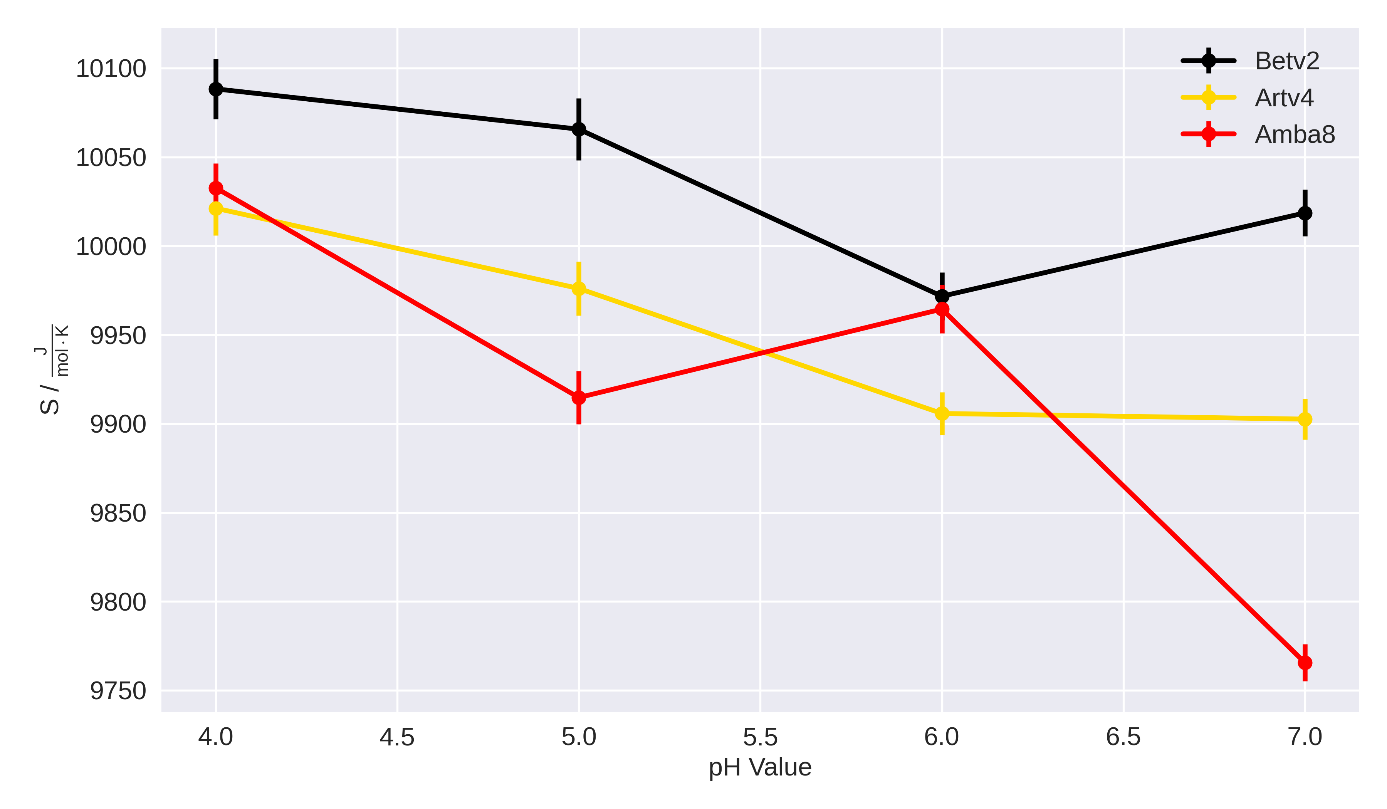


SI Figure S3: Overall sum of the residue-wise dihedral entropies at each pH value for all investigated profilin allergens Higher values of S denote a higher flexibility of the respective allergens.

.
